# Study on the Mechanical Properties of Two General-Purpose Cement–Lime Mortars Prepared Based on Air Lime

**DOI:** 10.3390/ma17051001

**Published:** 2024-02-22

**Authors:** Armando Zagaroli, Jan Kubica, Iwona Galman, Kristian Falkjar

**Affiliations:** Department of Structural Engineering, Faculty of Civil Engineering, Silesian University of Technology, 44-100 Gliwice, Poland; armando.zagaroli@polsl.pl (A.Z.); iwona.galman@polsl.pl (I.G.); kristian.falkjar@polsl.pl (K.F.)

**Keywords:** air lime mortars, flexural strength, compressive strength, fracture energy, split-cylinder tensile strength

## Abstract

It is believed that the use of mortars based on air lime in the construction and renovation of brick buildings has a number of advantages, especially those closely related to the durability and strength of the structure. However, there is still a noticeable difference in the mechanical properties of these materials. This research investigated the mechanical characteristics of a mixed cement–lime mortar with the two most popular proportions of an air lime, cement, and sand mix: 1:1:6 and 1:2:9 (by volume). Mechanical tests were performed on standard and non-standard samples to assess compressive strength, tensile strength, flexural strength, and fracture energy. The obtained results indicate the possibility of using these mixtures in modern masonry construction, as well as in the aspect of sustainable development. Additionally, lime mortar with a higher lime content can be used in non-load-bearing walls and in renovation and repair works.

## 1. Introduction

Lime mortars were widely used in masonry construction as the predominant mortars until the early 20th century, when they were replaced by mortars with Portland cement as a binder component. This change was primarily due to the higher strength of mortars with the addition of cement, as it was widely known that pure lime–sand mortars had lower mechanical strength [1,2,3]. Nevertheless, it was recognized that the use of lime and cement-based air lime mortars could be beneficial for various aspects, especially those closely related to the durability of masonry structures. These include enhancing durability against freeze–thaw cycles, improving mortar workability, and increasing resistance to water penetration [4,5,6]. Furthermore, based on the works by Campo et al. [7,8], mixed air lime–cement mortar, although to some extent limited compared to pure air lime mortars, is subjected to the carbonation process, aimed at reducing CO2 air content with gradual strength gain. Considering all these aspects, there is potential value in rediscovering the mixing of these two binders as a means to tackle the trade-off between their inherent limitations. It is worth noting that only recently has sustainability started to be taken into account in comparative life cycle assessments (LCA) of different binders at cradle-to-cradle level [9].

Despite the recent increasing interest in utilizing air lime mortars in masonry buildings (including historical structures), their effective reuse has faced challenges in their applications because of various factors, including a lack of scientific and technical knowledge even for historical building applications [10]. In this context, natural hydrated lime (NHL) is primarily employed in plaster and render coatings of masonry surfaces, and significant distinctions in behavior should be considered when conducting tests on mortars with low or high mechanical strength in relation to their applications [11,12]. Specifically, characterization techniques, focusing on comprehending the chemical, mineralogical, and petrographic properties [13,14,15] along with indirect measurements and tests on non-standard samples for the mechanical properties [16,17], are commonly employed in heritage masonry structures with soft mortars to identify suitable materials for conservation purposes. Additionally, numerous applications in this area focus on pure air lime [18] and hydraulic lime, which is also considered compatible with masonry material. In their research, Lanas et al. [19] specifically examined the connections between the binder/aggregate ratio and the characteristics of various aggregates concerning the strength and porosity of natural hydraulic mortars. They demonstrated that altering the granulometry allows for the attainment of improved strength with grain size distributions that exclude rock fragments. Additionally, mortars with higher binder content exhibit superior compressive and flexural strengths, but unlike cement-based mortars, show an increase in open porosity. Apostolopoulou et al. [20] have also investigated the significance of choosing a suitable binder-to-aggregate ratio, as well as the consistency and type of natural hydraulic lime (NHL), in view of a performance-based approach. Jaafri et al. [21] conducted an analysis of blended mixes incorporating hydraulic lime and cement, also investigating their long-term drying shrinkage in contrast to pure cement mortars, with a positive outcome for the addition of hydraulic lime in the binder. However, Silva et al. [22] have emphasized that for historical masonry applications, the cement content should exceed 25% to achieve early-age strength enhancement but should remain below 50% to minimize the risks of incompatibility. The same authors in another work [23] showed that a mix of natural hydraulic lime and air lime, with hydraulic content superior to 25%, can overcome certain drawbacks of aerial lime mortars, such as prolonged hardening times, making them more durable without making them excessively incompatible with historical materials. More recently, solutions of mixed hydraulic lime and cement mortars are being mechanically enhanced with short fiber additions [24,25,26] for existing masonry retrofitting operations with the objective of furnishing materials with more rapid applications compared to the most common fabric-reinforced cementitious matrices and textile-reinforced mortars (FRCM-TRM) [27,28]. However, the testing of the effectiveness of fiber-reinforced lime-based mortars in improving the in-plane masonry behavior at the macro-scale is still limited [29,30].

Particularly referring to blends comprising aerial lime and cement, the studies performed by Arandigoyen et al. [31] and D’Ayala et al. [32] showed that mortars with air lime could develop a ductile behavior compared to the cement, despite the latter possessing higher strength but more brittle behavior. Vasovic et al. [33] observed that the substitution of lime with 20 wt% of white Portland cement in a mixture, combined with a reduction in the water-to-binder ratio and the inclusion of an air-entraining agent, improves mortar strength without affecting porosity. This outcome is credited to the air-entraining agent, which facilitates the incorporation of CO_2_ into the material’s structure. However, further studies are needed to delve deeper into this phenomenon. From a masonry mechanical point of view, it has already been observed how mortars with low mechanical strength can result in masonry with higher compressive strength than masonry made with mortars with high mechanical strength [34,35], but the experimental research focused on air lime cement mortars is limited in terms of multiscale masonry performance [36]. In this context, Ramesh et al. [37,38] investigated the impact of various lime–cement volumetric proportion ratios on both the mechanical properties of mortars and the shear bond strength on clay triplets. In line with Alecci et al. [39], the observed higher values of compressive strength in mortars are associated with an increase in the initial shear strength. In another work, the same authors [40] formalized linear functions of the compressive and flexural strength, incorporating parameters such as lime content in the binder, binder aggregate ratio, and curing age. Costigan et al. [41] observed how lime-based mortars have the potential to achieve a greater bond strength compared to hydraulic mortars and masonry constructed with calcium lime mortar CL90, exhibiting a high wrench bond strength, and are stronger than masonry built with a mortar of elevated hydraulic strength but weaker bond strength. Sarangapani et al. [42] previously noted a strong correlation between masonry compressive strength and bond, irrespective of the mortar compressive strength. At the masonry level, Costigan at al. [43] tested the compressive strength of pure hydrated lime mortars and masonry at different times, showing the highly nonlinear stress–strain behavior of the compressive strength with the increasing lime content and how EN 1996-1-1:2005 [44] models predict with increased precision the experimental results after 6 months. Comparisons of the performance of pure aerial lime masonry in standard and non-standardized tests have also been performed in Pelà et al. [45,46], for deepening the knowledge of the behavior of historical masonry structures. Brando et al. [47] proposed a multi-scale approach for a finite element method (FEM) numerical model of historical masonry based on air lime. This approach involves the customized preparation of an air lime mortar based on previous chemical analysis and the validation of a continuous numerical FEM model by means of tests conducted on a limited amount of masonry triplets, assembled with bricks and the previous mortar.

In this study, experimental characterizations of two common mixes of air lime cement mortars with two different lime–cement mix proportions are carried out. Compressive strength of prismatic and cylindrical samples, flexural strength on standard prisms, and split-cylinder tensile strength of cylindrical samples were determined. Values of the fracture energy at 75 days, determined based on the three-point bending test on notched beams, are also obtained both on measurements of the vertical beam deflections and the crack mouth opening displacements. The information obtained on the mechanical properties from testing specimens of different shapes can be useful for disseminating these mortar mixes in engineering practice and for applications in the micro-modeling of masonry made with the usage of these types of mortars [48].

## 2. Materials

**Figure 1:**
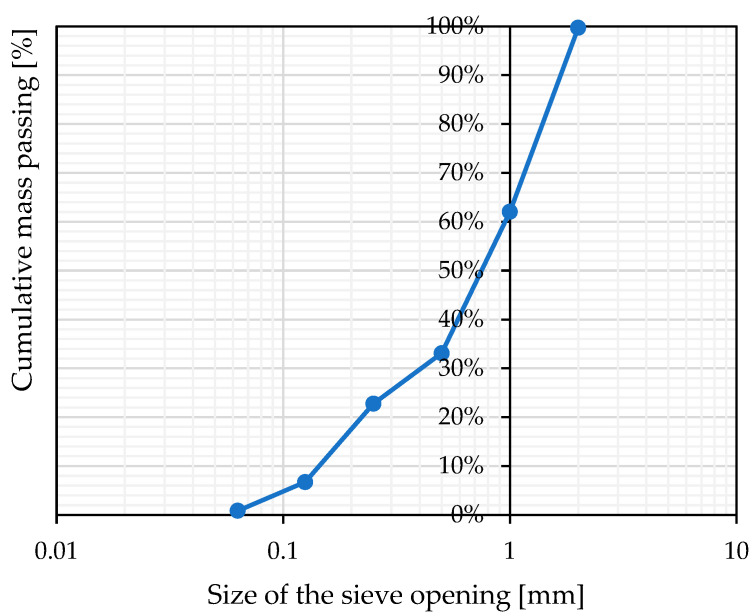
Adopted sand granulometry.

Air lime CL90-S [49] and Portland cement CEM II/B-32.5 R [50] were selected as binders’ components for the mortar mixes. Siliceous sand of grading 0/2 mm was chosen as aggregate for the mixes (Figure 1). Two mortar mixes were considered with volumetric proportions of cement, lime, and sand of 1:1:6 and 1:2:9, namely, MIX-1 and MIX-2. Measurements of the masses of the components based on the volume proportions and bulk densities evaluated are given in Table 1. The water content in the mix is a crucial parameter that must be considered, as a high amount of water can lead to a reduction in mechanical properties [51]. The water–binder ratio was calibrated based on the flow table test in accordance to EN 1015-3 [52]. In order to ensure practical usability of the mortars by masons in real-world applications, a wide slump range of 175 ± 10 mm was maintained. Specifically, for MIX-1 and MIX-2, the resulting slumps considered acceptable were 170 mm and 166 mm, respectively. For each mortar mix analyzed, specimens were cast after vibration, since different compaction methods have not showed any particular influence on the mechanical strength of mortars [53], and cured in the same conditions. Specifically, mortar specimens were kept at a stable temperature of 20 ± 2 °C and humidity conditions of 65% ± 5% for 21 days after the previous 7 days of curing in polyethylene bags, as specified in the European standard EN 1015-11 [54], except the samples for fracture energy testing, which were kept in the climatic chamber for 75 days. The experimental program of the tested series is given in Table 2, along with the curing conditions of each series. In Table 2, the individual test series are identified by a code separated by an underscore. The first part of the code indicates the type of test: BT for small beam specimens subjected to a three-point bending test, CT for half of small beams subjected to a compression test, CCT for cylindrical specimens subjected to a compression test, SCT for cylindrical specimens subjected to a Brazilian split-cylinder test, and FT for cuboidal beams used to determine fracture energy. The second code number recalls the volume proportions of the two mixtures: 116 for MIX-1 and 129 for MIX-2.

## 3. Methods and Test Set-Up Details

Mechanical tests were carried out on cured specimens according to the selected storage procedure. A summary of the tests carried out with respect to the predefined test series, standard marking, shape dimensions, and number of specimens considered is given in Table 3.

### 3.1. Flexural and Compressive Strength on the Basis of EN 1015-11 [54] 

**Figure 2:**
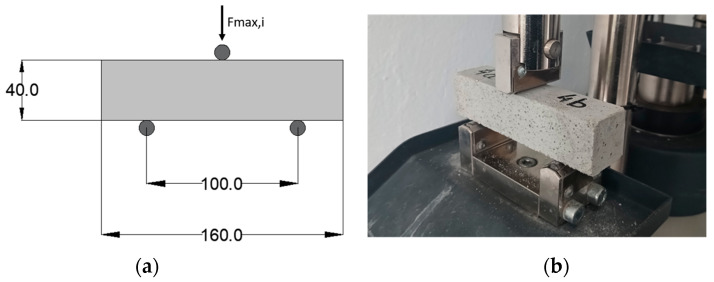
(**a**) Flexural tests on a standard prismatic sample with static scheme and dimensions in mm and (**b**) view of the typical specimens positioned in the testing machine, ready for examination.

First, three-point bending tests were conducted on mortar small beam specimens measuring 40 mm × 40 mm × 160 mm, in accordance with EN 1015-11 [54]. The samples were subjected to a concentrated compressive force, acting at the midpoint of the span, progressively increasing until reaching the point of failure. The distance between the support points was standardized at 100 mm. Six specimens for each mix were tested in load control with a load rate of 0.05 kN/s. Figure 2a illustrates the applied loads in the static diagram and the geometry of the tested elements, while Figure 2b depicts the typical element prepared for testing in the machine.

**Figure 3:**
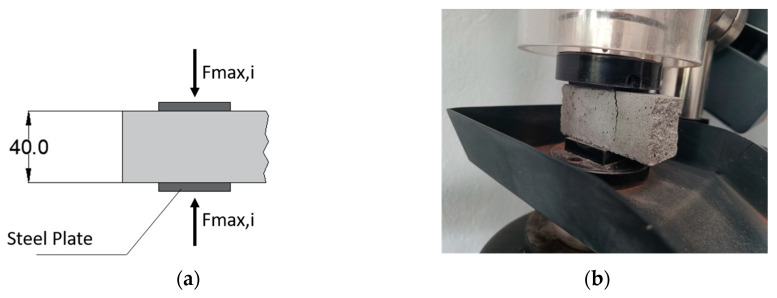
(**a**) Compressive tests on half-small beams’ sample with static scheme (dimensions in mm) and (**b**) view of the standard specimens placed within the testing apparatus.

In accordance with the previous specifications outlined in EN 1015-11 [54], the assessment of the compressive strength of the mortar using the resulting halves of small beams obtained from bending tests was undertaken. The axial compression was guaranteed, utilizing square-shaped steel plates with dimensions of 40 mm × 40 mm and applying a uniform vertical load with the same load rate of the previous test. Figure 3a illustrates the static scheme with dimensions of the compressed half-beam elements while Figure 3b illustrates a representative sample in the machine.

### 3.2. Compressive Strength and Stress–Strain Relationship on the Basis of the Cylinder Specimens Test 

**Figure 4:**
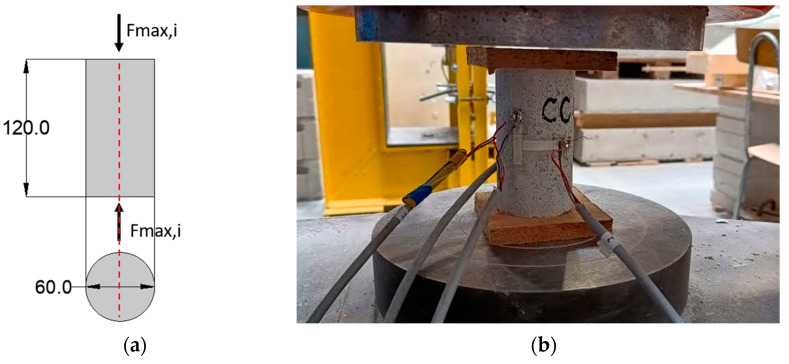
(**a**) Static scheme of compressive tests on a cylindrical sample with dimensions in mm and (**b**) specimens placed within the testing apparatus with detail of measurement system.

Compressive tests were also carried out on cylindrical specimens with 60 mm of diameter and 120 mm height (Figure 4a). In particular, a displacement control test set-up was used for the cylindrical compressive tests with a load rate of 0.05 mm/s, for investigating the post-peak softening behavior. In this way, these tests are intended to be destructive tests where in the pre-peak behavior, recommendations of EN 12390-13 [55] for hardened concrete were taken into account for the determination of the elastic modulus, according to the ratio of the stress and strain between 30% and 50% of the peak values. Axial and transversal deformations were measured with four strain gauges placed at the center of the lateral cylinder surface, for the evaluation of Poisson’s ratio (Figure 4b).

### 3.3. Tensile Strength on the Basis of ASTM C496 [56] 

**Figure 5:**
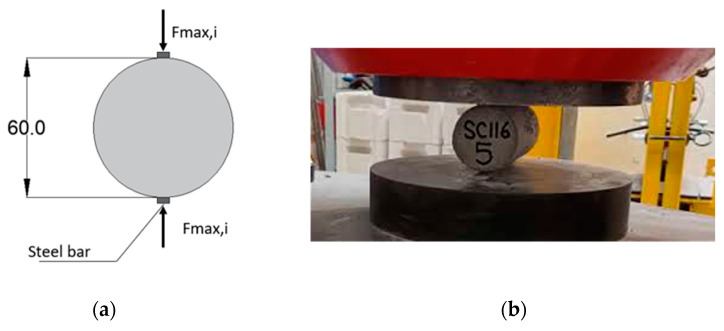
(**a**) Static scheme of split-cylinder tensile strength on a cylindrical sample with dimensions in mm and (**b**) testing set-up adopted.

Tensile tests with split-cylinder were also carried out on cylinders in accordance with ASTM C496 [56] with the dimensions of the specimens used for compression tests (Figure 5a). For these tests, a displacement control system operating at a loading rate of 0.01 mm/s was selected, in which the application of the split-cylinder load by the actuator was realized by means of a flat steel bar of the same length of the cylindrical specimens (Figure 5b).

### 3.4. Fracture Energy Test 

**Figure 6:**
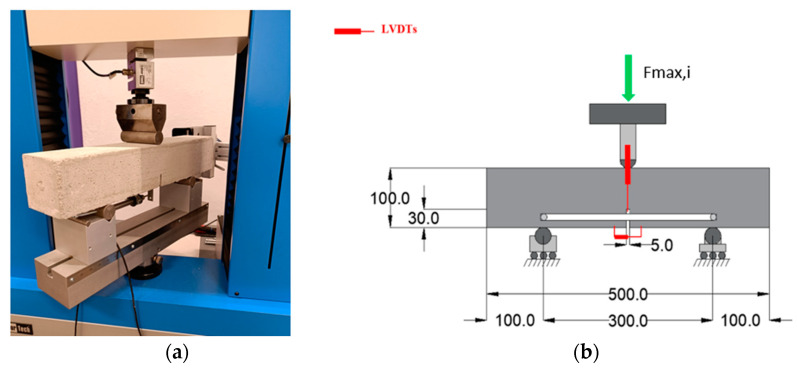
(**a**,**b**) Fracture energy test set-up with dimensions (mm).

Measurements of the fracture energy were carried out by means of three-point bending tests on notched beams with dimensions 100 mm × 100 mm × 500 mm, in line with RILEM FMC-50 [57]. The dimensions of the notch are 5 mm in thickness and 30 mm in depth. The depth of the notch was chosen based on Hillerborg’s recommendation [58], which suggests using a notch with main dimensions ranging from 0.3 to 0.4 of the depth of the beam. The specimens were subjected to loading at a steady displacement rate of 0.1 mm/min. The tests were conducted utilizing a hydraulic testing machine with a capacity of 5 kN, which allowed for precise control over the displacement. From the same test set-up, measurements of crack mouth opening displacements (CMOD) were obtained using another clip gauge in correspondence of the notch (Figure 6a,b). This allowed a comparison of the values of fracture energy based on mid-span deflection of the beams and based on CMOD, according to the proposal of Japan Concrete Institute (JCI-S-001–2003) [59]. More precisely, the proposals of RILEM FMC-50 (1) and JCI-S-001–2003 (2) share formal uniformity, differing only in their respective approaches to deflection, crack mouth opening displacement, and a multiplying coefficient:

(1) $$G_{f-\delta }=\frac{\left(W_{\delta }+\frac{mS}{L}g\delta \right)}{\left(d-a\right)b}$$

(2) $$G_{f-CMOD}=\frac{0.75\;(W_{CMOD}+\frac{mS}{L}\;g\;CMOD)}{\left(d-a\right)b}$$

where *W_δ_* and *W_CMOD_* are the areas under the load–deflection and load–CMOD curves, respectively; *mS/L* is the weight of the beam between the supports where *m* is the total weight of the beam, and *S* and *L* denote the length of the support and the total length of the beam; g is the gravity acceleration; *δ* and CMOD are the deflection and crack mouth opening displacement at the final failure of the beam; *d* is the beam height; *b* is the beam width; and *a* is the notch depth.

## 4. Test Results

The results of the experimental investigations are depicted in both graphical and tabular formats with descriptions of relative failure modes. Table 4 presents the results for average flexural strength on six standard small beam specimens and compressive strength on the resulting twelve half-beams for each mortar mix. As expected, the higher air lime content leads to the reduction in the mechanical properties. For mortar type MIX-1, this results in a compressive strength of 7.91 MPa (13.1%) and a flexural strength of 2.21 MPa (16.2%). Meanwhile, mortar MIX-2 exhibits 4.16 MPa (7.6%) for compression and 1.23 MPa (11.2%). The values of the coefficient of variation (CoV) are given in parentheses. In tests conducted on half-beams (prism specimens), the compressive strength of the MIX-2 mortar is approximately 52% of the strength determined for the MIX-1 mortar with half lime content in the binder. A similar relationship occurs in the case of flexural strength. The MIX-2 mortar achieved approximately 55% of the strength of the MIX-1 mortar. Surprisingly, there were also lower CoV values in the case of mortars with a higher lime content in the binder (MIX-2), despite the fact that the tests were carried out using the same devices and with equal care and control. 

**Figure 7:**
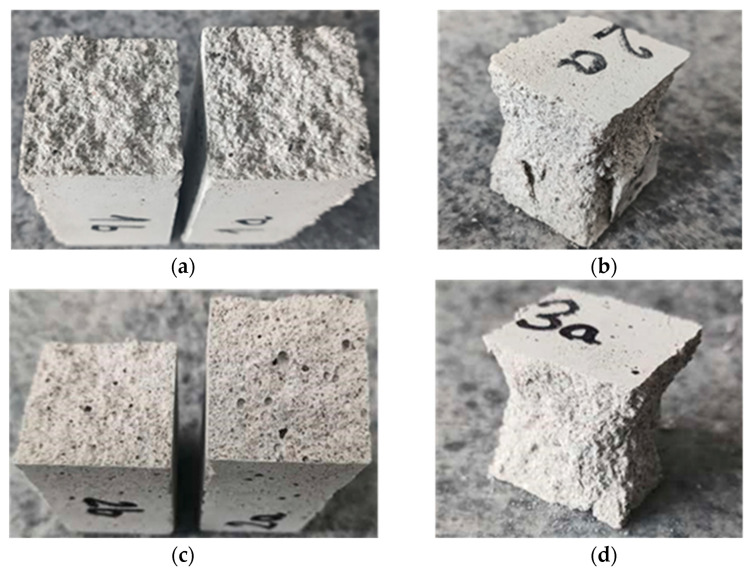
(**a**) Typical failed samples in three-point bending tests and (**b**) compressive loads for mortar MIX-1 (1:1:6). (**c**) Example of failed specimen in three-point bending test and (**d**) compressive loads for mortar MIX-2 (1:2:9).

The failure modes in the two loading conditions were consistent for both mortar mixes not being influenced by the different lime contents in binder composition. In the three-point bending test, crack development occurred in the middle beam cross-section (Figure 7a–c), while half-beams failing in compression exhibited a conical shape failure with the expulsion of material from the lateral surfaces at failure (Figure 7b–d). The fractures also displayed a notable quantity of observable pores with more evident porosity for the samples made with higher lime content (Figure 7c). This can be attributed to the presence of air lime, which functions as a natural air-entraining additive, enhancing workability and frost resistance while simultaneously diminishing strength.

**Figure 8:**
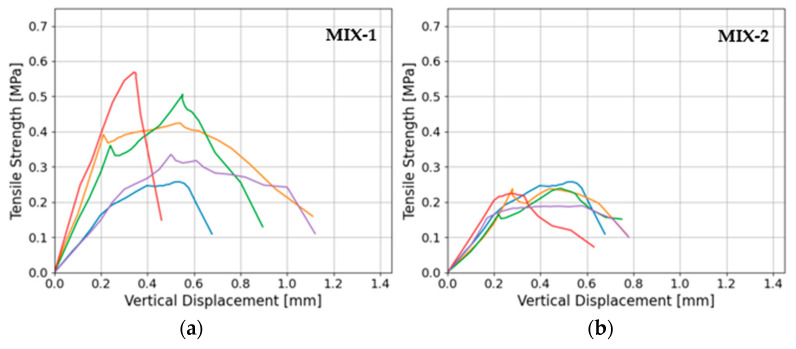
(**a**) Tensile stress–vertical displacement curves in split-cylinder tests for MIX-1 and (**b**) for mortar MIX-2. Different colors lines represent different samples.

**Figure 9:**
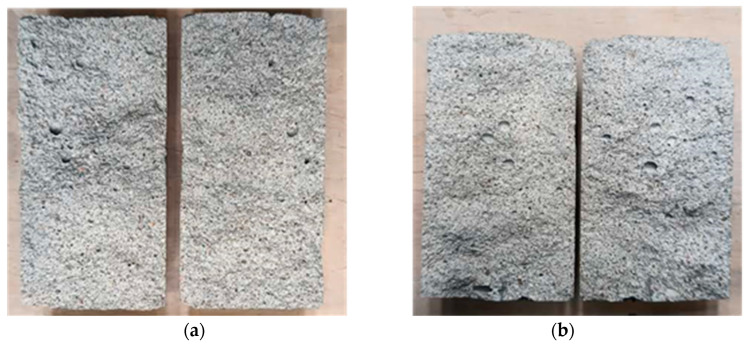
(**a**) Examples of failed samples in split-cylinder tensile strength tests for MIX-1 and (**b**) for mortar MIX-2.

Results of the split-cylinder tensile strength on five cylindrical samples for each mix show peak mean values of 0.46 MPa (19.1%) and 0.23 MPa (11.0%). Similarly to the compressive and bending strength determined on the beam halves, in the splitting test of cylindrical samples, the tensile strength determined in this method for the mortar with a higher lime content (MIX-2) was twice lower than in the case of the MIX-2 mortar. Moreover, the dispersion of results, and therefore of CoV, was almost twice as large in the case of the MIX-1 mixture (with a lower lime content in the binder). The post-peak tensile behavior of both mortar mixes demonstrates a ductile characteristic (Figure 8a,b). In any case, the failure modes exhibited uniformity across all analyzed samples, featuring the typical separation of the cylinders into two parts along their diameter (Figure 9a,b).

**Figure 10:**
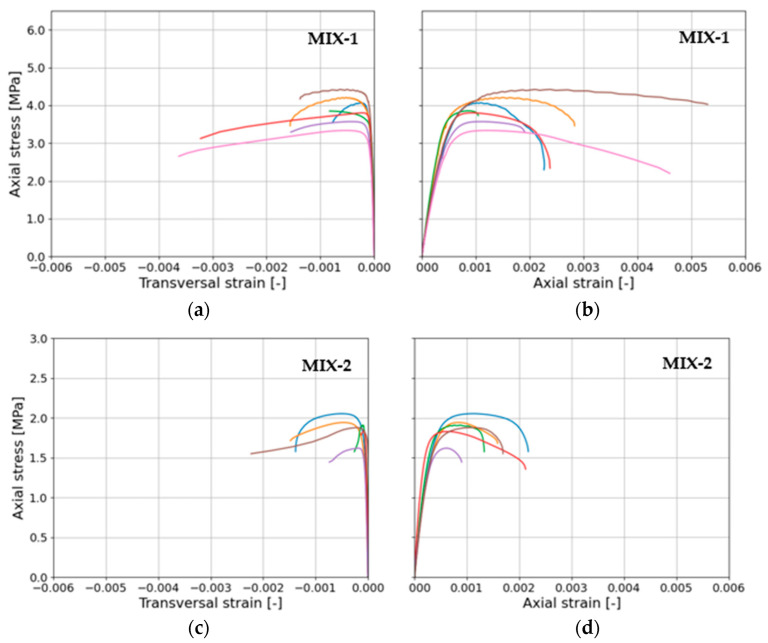
Compressive stress–strain relationships from cylindrical testing. (**a**) Axial compressive stress vs. transversal strain and (**b**) axial compressive stress vs. axial strain for MIX-1. (**c**) Axial compressive stress vs. transversal strain and (**d**) axial compressive stress vs. axial strain for MIX-2. Different colors lines represent different samples.

Figure 10a–d depict the results of compressive axial stress–axial/transversal strains for seven cylindrical samples for MIX-1 and six samples for MIX-2.

Consistent with the tests conducted on standard prisms (halves of beams after flexural tests), materials with lower lime content exhibit higher compressive strength. Analyzing the graphs shown in Figure 10, it can be seen that the mortar with a higher lime content in the binder (MIX-2) exhibited more brittle behavior. Compared to the MIX-1 mortar mixture (the content of lime in the binder composition was halved), the MIX-2 mixture showed, in addition to the more than twice lower value of maximum compressive stresses (compressive strength), slightly lower horizontal and vertical maximum strains (at the moment destruction). The results in terms of average parameters determined from these investigations are given in Table 5. Particularly, assessing material post-peak behavior, a value of the ductility (*μ*) is derived from an elastic–plastic bilinearization of the stress–strain relationship for each mix. The bilinearization adopted involves establishing a secant stiffness at 0.75 of the peak compressive strength, the maximum peak compressive strength, and ultimate deformation by ensuring the equivalence of the area under the bilinear curve with the obtained stress–strain relationship. Additionally, the assessment includes elastic properties, namely the peak cylindrical compressive strength (*f_cc_*), elastic modulus (*E_c_*), and Poisson’s ratio (*ν*).

**Figure 11:**
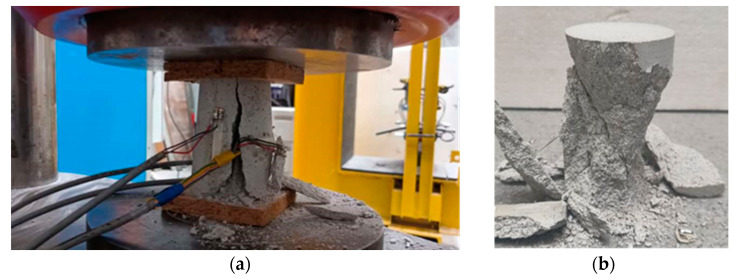
(**a**) Compressive failure of cylindrical samples for MIX-2 with tensile cracks along the height of the cylinders; (**b**) conical failed sample of mortar MIX-1.

Failure modes for both mixes typically comprised three types: conical, tensile cracks with their development spanning all the samples, and a mix of the previous, resulting in complete desegregation of the specimens at the point of failure. In the mix with higher lime content (MIX-2), tensile cracks were generally evident representing the typical failure, whereas for mortar MIX-1, conical and mixed failure modes were also identified (Figure 11a,b), therefore typical of what is observed in tests of cement mortar and concrete samples. 

A comparison of the compressive strength values of both types of mortars determined on the samples made from beam halves (*f_c_*) tested for bending (prism specimens) and on cylinders with a diameter of 60 mm and a height of 120 mm (*f_cc_*)—the scale and shape effect—is as follows:

- for MIX-1 mortar: *f_cc_*/*f_c_* = 0.49;
- for MIX-2 mortar: *f_cc_*/*f_c_* = 0.45.

**Figure 12:**
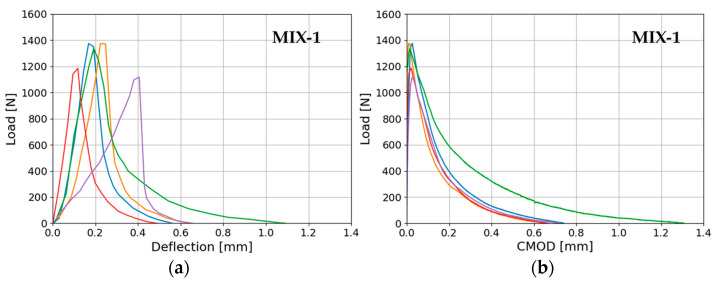

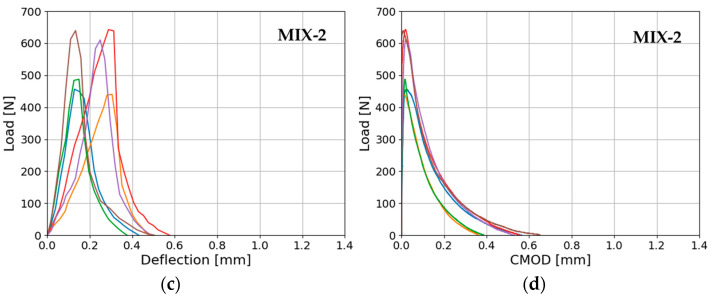
Load–displacement curves from three-point bending tests: (**a**) load vs. mid-deflection for MIX-1, (**b**) load vs. crack mouth opening displacement of the notch (CMOD) for MIX-1, (**c**) load vs. mid-deflection for MIX-2, and (**d**) load vs. (CMOD) for MIX-2 (1:2:9). Different colors lines represent different samples.

Figure 12a–d illustrate the load–deflection/CMOD curves for its fracture energy evaluation. These curves are related to five and six notched beams for MIX-1 and MIX-2, respectively. It is important to note that the influence of the beam weight between the supports has been excluded from the plots. Consequently, the area under the curves solely reflects the quantities *W_δ_* and *W_CMOD_* as per Equations (1) and (2). The test was carried out until complete failure due to fracture of the ligament and complete separation in the middle of the beam halves, on surfaces that generally were irregular. The trend reveals higher values of fracture energy for the mix with a lower amount of air lime. Furthermore, the results indicate higher values for fracture energy based on deflection (1) compared to that based on CMOD (2) for both mixes (Table 6).

**Figure 13:**
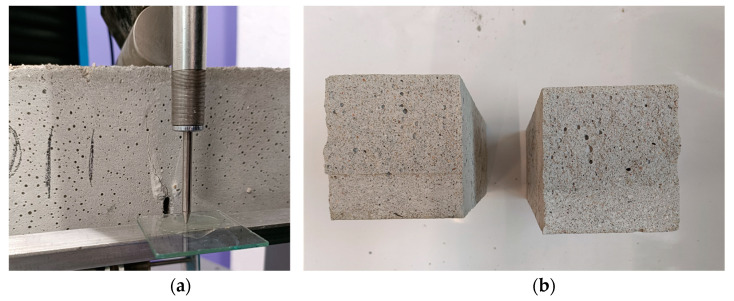
(**a**) Fracture process of the sample during the test with irregular surface. (**b**) Example of failed sample after the test with MIX-1.

The failure mode observed in the tests remained consistent with those obtained in the flexural tests, due to the similarity of the static scheme. The fracture path and growth did not vary between the two mixes. However, the fracture shape at the fracture zone was not smooth and did not always follow on a straight line with the middle beam cross-section (Figure 13a). Figure 13b provides a visual representation of failed samples for both mortar mixes, showcasing the irregularity of the fracture and the cross-sectional voids compared to the notch cross-section saw-cut one day before the test.

## 5. Discussion

**Figure 14:**
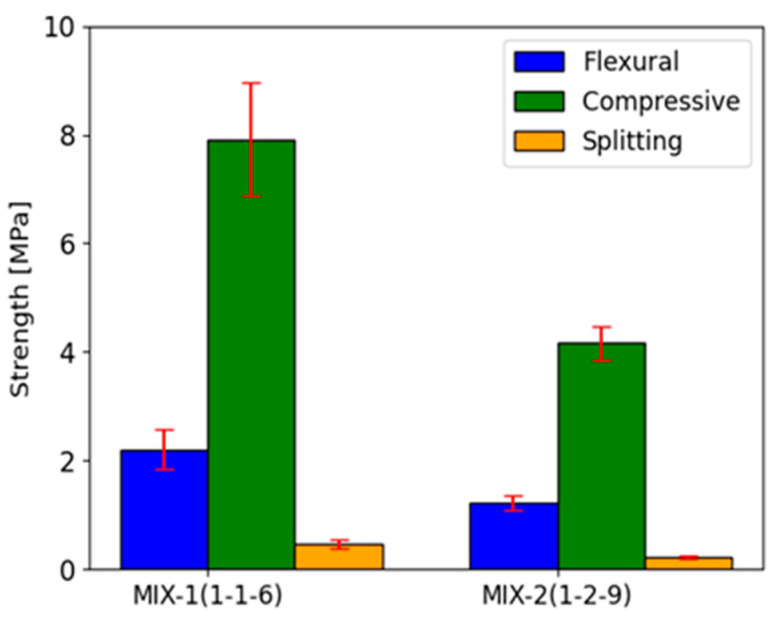
Comparative results for mortar mixes in terms of flexural, compressive, and split-cylinder strength.

Increasing the addition of lime in the binder composition is associated with a change in the compressive, tensile, and bending strength of the mortar. In the presented tests, there is an approximately 50% reduction in the compressive, flexural, and split-cylinder strength between the mortar with a higher lime content (MIX-2) and the mortar with a lower lime content (MIX-1) (Figure 14). However, the increased presence of lime in the binder is connected to the mix’s capability of sustaining deformation during the inelastic phase, leading to a difference of 9.34% in the obtained ductility values for the mixes. 

This contributes to the potential use of lime-based mortars in structural masonry, considering that the presence of lime can positively influence the behavior of the masonry once compressive strength has been achieved, as also assumed by Lumantarna et al. [60] based on experimental tests, both in the laboratory and with existing masonry materials. From a code standpoint [44], mortar MIX-1 can be categorized as an M5 mortar, achieving a minimum compressive strength of 5 MPa at 28 days. This allows for the use of this mortar in masonry operations also involving load-bearing capacity elements. As for Mortar MIX-2, which closely resembles an M5 mortar with approximately 50% lime (by mass) in the binder, it is suggested for masonry applications that do not require checking the ultimate limit state and for repair works on existing structures [22], provided there is a prior evaluation of the risk associated with the soluble salt content.

The dependencies between the ratios of mean peak flexural (*f_l_*) to compressive strength (*f_c_*), split-cylinder (*f_t_*) to flexural (*f_l_*) strength, and split-cylinder (*f_t_*) to compressive (*f_c_*) strength on half-beams are given in Table 7 against the lime content in the binder composition (33.8% for MIX-1 and 50.5% for MIX-2 by mass). It is possible to notice how the lime content does not influence the ratio of the compressive and split-cylinder strength that remains constant at 0.06, close to literature values [61]. It is noteworthy that direct measurements of the tensile strength of masonry mortars are infrequent in the literature. Typically, indirect measurements derived from flexural strength are provided, relying on concrete relationships, as seen in the MODEL code [62]. Furthermore, the ratio of split-cylinder tensile strength to flexural strength diminishes with an increase in lime content in the binder, indicating a more pronounced reduction in split-cylinder tensile strength compared to flexural strength when air lime is added to the binder. Conversely, the ratio of flexural strength to compressive strength rises, suggesting an accelerated decline in compressive strength as the air lime addition increases. Nevertheless, these variations should be considered as initial indicators and should be enhanced with additional lime content information.

Regarding the compressive strength of the cylinders, the consistent difference in compressive strength between cylinders and beams revealed average ratios, with the cylindrical compressive strength to half-beam compressive strength ratio, at 0.49 and 0.45 for MIX-1 and MIX-2, respectively. These low values can be ascribed, partially, to differences in the slenderness of prismatic and cylindrical samples, along with variations in the load and displacement control test set-ups employed for each type. However, a major contributing factor is due to the incorporation of soft plywood plates (Figure 4b) between the steel plates and cylindrical samples. This addition significantly reduced friction, consequently leading to a reduction in strength with a similar impact observed when capping top and bottom surfaces with sliding materials like Teflon [63]. Based on the experimental data acquired for Poisson’s ratio, it appears that this aspect remains unaffected. The observed Poisson’s ratios align with the values commonly reported in the literature for lime, cement, or blended mortars, falling within the range of 0.15 to 0.25 [64]. The same consideration can be performed for the elastic modulus showing respect to literature values from similar investigations [37]. In this scenario, expected values of the porosity for the mixes can be also found in Ramesh et al. [40], who examined air lime-based mortars with identical proportions by means of measurements of percentage of pores accessible to water. Open porosity according to [40] follows a decrement with increasing curing age and an increment for increasing lime contents in the binder, expecting at 28 days 24.2% and 26.0% for mixes with 50% and 66.7% of lime content in volume, respectively.

In case of fracture energy, both values computed based on deflection and CMOD are comparable, with a slightly lower fracture energy based on CMOD with a reduction in the fracture energy based on deflections of 32.8% and 31.7% for MIX-1 and MIX-2, respectively. The primary cause of this difference lies in the inclusion of the multiplying coefficient of 0.75 in the JCI-S-001–2003 [59] formulation, with a relatively minor influence from the area under the load–deflection and CMOD curves, where the superior area under the load–deflection curve compared to the CMOD area was also noted in other research studies [65]. Both values of fracture energy align with those obtained by Ramesh et al. [37] in tests involving mortar mixes with the same volume proportions and calcium lime, but using CEM I 42.5 R. Their experiments [37] included subjecting standard small beam samples, measuring 40 mm × 40 mm × 160 mm, to three-point bending and measuring the deflection. In this context, the higher fracture energy values observed in our investigations, as per RILEM [57], apart from the material differences, may be linked to specimen size issues (scale effect) [66]. Specifically, larger beam sizes tend to exhibit higher values of fracture energies according to this approach [67]. Regarding this phenomenon, in the context of masonry mortar characterization, various authors suggest using smaller sample dimensions for assessing fracture energy [68,69]. To address the influence of reduced specimen sizes, adjustments to the RILEM formulation were also recommended [70]. These adjustments consider the area beneath the load–deflection curve’s tail, employing a hyperbolic discretization method instead of considering the weight of the beams. The presented study was focused more on the comparison of the CMOD and RILEM and the latter has been disregarded for the application of standard formulation. Finally, Table 8 summarizes the mean and coefficient of variation for standard values of the flexural strength, fracture energy, tensile strength, and compressive strength computed for the mixes.

## 6. Conclusions

The paper presents the results of experimental research investigating the influence of the air lime content in the binder composition of cement–lime mortars on their basic mechanical properties. In view of this objective, two of the most characteristic mortar mixes with different air lime contents are examined. In this case, these mortar mixes were prepared using lime content comprising 34% and 50% of the total binder mass, maintaining volumetric proportions typical for bricklaying, namely 1:1:6 and 1:2:9 (Cement:Air lime:Sand). The focus was on their mechanical parameters, encompassing bending strength, compressive strength, split-cylinder tensile strength, and fracture energy. To assess the nonlinear softening characteristics of the mixes, compressive strength tests were conducted both on cylinders and on standard beams’ shape specimens. Considering the presented analysis of the test results presented above, the following conclusions can be drawn:

- Mortars with a higher content of lime in the binder show a deterioration in their mechanical properties, with the exception of ductility and Poisson’s ratio. As a result, the material is softer but can withstand greater deformation and expansion, which can be seen as a positive aspect (reduction of cracks) in masonry structures. In the presented tests, the compressive strength of the mortar with the ingredient ratio of 1:1:6 (MIX-1) was almost higher than that obtained for the mortar 1:2:9 (MIX-2);
- Analyzing the dependencies involving the ratios of standard compressive, flexural, and split-cylinder tensile strength, a more pronounced decline is evident for compressive and tensile strength compared to flexural strength. However, these findings should be viewed as initial insights, and further mechanical investigations with different lime contents should be taken into account;
- Obtained values of the modulus of elasticity, Poisson’s ratio, compressive strength, flexural strength, and fracture energy are in line with the literature values for mixes with the same volumetric proportions and air lime but different cement types. A summary is given in Table 8;
- Differences for the compressive strength values from cylinders and standard beam specimens could be ascribed to the different shape and overall dimensions of the specimens, and to different top and bottom capping used, whereas difference in the evaluation of the fracture energy based on two different standard approaches are more related to the differences in the coefficients in their formulations.

Further investigations should explore diverse mortar mixes incorporating varying lime contents for examining the dependency on diverse lime-to-binder and binder-to-aggregate ratios. To assess the compatibility of these mixes with the parameters of existing masonry (e.g., in cases of historical constructions), additional analysis should focus on porosity distribution and the shape of voids, utilizing imaging techniques such as Micro-CT, alongside chemical and morphological studies. These aspects will be the subjects of future research.

## Figures and Tables

**Table 1:** Mortar compositions.

Mixes’ Composition (Cement:Air Lime:Sand) by Volume	Cement [g]	Air Lime [g]	Sand [g]	Water/Binder (by Mass)
MIX-1 (1:1:6)	153	78	1350	0.92
MIX-2 (1:2:9)	102	104	1350	1.04

**Table 2:** Testing protocol with specifications for storing specimens.

Mixes’ Composition (Cement:Air Lime:Sand) by Volume	Testing Series	Total Curing Time (Days)	Storage Time at Temperature of 20 ± 2 °C and Relative Humidity 65 ± 5%		
			In the Mold and Polyethylene Bags	Without the Mold in Polyethylene Bags	Without the Mold and Polyethylene Bags
	BT_116–CT_116	28	2	5	21
MIX-1 (1:1:6)	CCT_116–SCT_116	28	2	5	21
	FT_116	75	2	5	68
	BT_129–CT_129	28	2	5	21
MIX-2 (1:2:9)	CCT_129–SCT_129	28	2	5	21
	FT_129	75	2	5	68

**Table 3:** Testing protocol with specifications for dimensions, number, and standard designation.

Mixes	Testing Series	Sample Shape (mm)	Number of Samples	Sample Size (mm)	Standard Remarks
MIX-1 (1:1:6)	BT_116	Small beams	6	40 mm × 40 mm × 160 mm	EN 1015-11 [36]
	CT_116	Half of small beams	12	40 mm × 40 mm × ~80 mm	EN 1015-11 [36]
	CCT_116	Cylinders	7	60 mm × 120 mm	EN 12390-13 (Elastic modulus) [37] and failure
	SCT_116	Cylinders	5	60 mm × 120 mm	ASTM C496 [38]
	FT_116	Cuboidal beams	5	100 mm × 100 mm × 500 mm	RILEM FMC-50 [39]
MIX-2 (1:2:9)	BT_129	Small beams	6	40 mm × 40 mm × 160 mm	EN 1015-11 [36]
	CT_129	Half of small beams	12	40 mm × 40 mm × ~80 mm	EN 1015-11 [36]
	CCT_129	Cylinders	6	60 mm × 120 mm	EN 12390-13 (Elastic modulus) [37] and failure
	SCT_129	Cylinders	5	60 mm × 120 mm	ASTM C496 [38]
	FT_129	Cuboidal beams	6	100 mm × 100 mm × 500 mm	RILEM FMC-50 [39]

**Table 4:** Flexural and compressive strength results.

Mixes’ Composition (Cement:Air Lime:Sand) by Volume	Flexural Strength [MPa] (CoV%)	Compressive Strength [MPa] (CoV%)
MIX-1 (1:1:6)	2.21 (16.2)	7.91 (13.1)
MIX-2 (1:2:9)	1.23 (11.2%)	4.16 (7.6)

**Table 5:** Compressive strengths of mortars determined from cylindrical samples.

Mixes	*f_cc_* [MPa] (CoV%)	*E_c_* [MPa] (CoV%)	*ν* [-] (CoV%)	*μ* [-] (CoV%)
MIX-1 (1:1:6)	3.89 (9.6)	7188.49 (5.1)	0.15 (19.2)	3.92 (29.2)
MIX-2 (1:2:9)	1.87 (7.7)	5078.12 (2.3)	0.19 (35.2)	4.28 (12.9)

**Table 6:** Test results of notched beams for the mean values of fracture energy.

Mixes	*G_f-δ_* [N/m] (CoV%)	*G_f-CMOD_* [N/m] (CoV%)
MIX-1 (1:1:6)	38.67 (28.6)	25.98 (8.0)
MIX-2 (1:2:9)	17.11 (22.8)	11.69 (24.5)

**Table 7:** Ratio of flexural (*f_l_*) to compressive (*f_c_*), tensile splitting (*f_t_*) to compressive (*f_c_*), and tensile splitting (*f_t_*) to flexural strength (*f_l_*) in dependence with the lime content of the two mixes.

Mixes	Lime Content by Volume (by Mass) [%]	*f_l_/f_c_* [-]	*f_t_/f_c_* [-]	*f_t_/f_l_* [-]
MIX-1 (1:1:6)	50.0% (33.8%)	0.28	0.06	0.21
MIX-2 (1:2:9)	66.7% (50.5%)	0.30	0.06	0.19

**Table 8:** Summary of the mechanical properties evaluated.

Properties (Standard)	MIX-1 (1:1:6)	MIX-2 (1:2:9)
Compressive strength (EN 1015-11)	7.91 MPa (13.1%)	4.16 MPa (7.6 %)
Flexural strength (EN 1015-11)	2.21 MPa (16.2%)	1.23 MPa (11.2%)
Compressive strength (on 60 mm diameter and 120 mm height cylinder specimens)	3.89 MPa (9.6%)	1.87 MPa (7.7%)
Split-cylinder tensile strength (ASTM C496)	0.46 MPa (19.1%)	0.23 MPa (11.0%)
Elastic modulus (EN 12390-13)	7188.49 MPa (5.1%)	5078.12 MPa (2.3%)
Poisson’s ratio	0.15 (19.2%)	0.19 (35.2%)
Ductility	3.92 (29.2%)	4.28 (12.9%)
Fracture Energy at 75 day (RILEM)	38.67 N/m (28.6%)	17.11 N/m (22.8%)

## Data Availability

The data presented in this study are available on request from the corresponding author. The data are not publicly available due to the unfinished SUBLime project.
